# Complement Lysis Activity in Autologous Plasma Is Associated with Lower Viral Loads during the Acute Phase of HIV-1 Infection

**DOI:** 10.1371/journal.pmed.0030441

**Published:** 2006-11-21

**Authors:** Michael Huber, Marek Fischer, Benjamin Misselwitz, Amapola Manrique, Herbert Kuster, Barbara Niederöst, Rainer Weber, Viktor von Wyl, Huldrych F Günthard, Alexandra Trkola

**Affiliations:** Division of Infectious Diseases, University Hospital Zürich, Zürich, Switzerland; Fred Hutchinson Cancer Research Center, United States of America

## Abstract

**Background:**

To explore the possibility that antibody-mediated complement lysis contributes to viremia control in HIV-1 infection, we measured the activity of patient plasma in mediating complement lysis of autologous primary virus.

**Methods and Findings:**

Sera from two groups of patients—25 with acute HIV-1 infection and 31 with chronic infection—were used in this study. We developed a novel real-time PCR-based assay strategy that allows reliable and sensitive quantification of virus lysis by complement. Plasma derived at the time of virus isolation induced complement lysis of the autologous virus isolate in the majority of patients. Overall lysis activity against the autologous virus and the heterologous primary virus strain JR-FL was higher at chronic disease stages than during the acute phase. Most strikingly, we found that plasma virus load levels during the acute but not the chronic infection phase correlated inversely with the autologous complement lysis activity. Antibody reactivity to the envelope (Env) proteins gp120 and gp41 were positively correlated with the lysis activity against JR-FL, indicating that anti-Env responses mediated complement lysis. Neutralization and complement lysis activity against autologous viruses were not associated, suggesting that complement lysis is predominantly caused by non-neutralizing antibodies.

**Conclusions:**

Collectively our data provide evidence that antibody-mediated complement virion lysis develops rapidly and is effective early in the course of infection; thus it should be considered a parameter that, in concert with other immune functions, steers viremia control in vivo.

## Abstract 2

Antibody-mediated complement lysis of HIV virions develops rapidly and is effective already early in the course of HIV infection.

## Editors' Summary

**Background.:**

If untreated, most people who become infected with the human immunodeficiency virus (HIV) eventually develop acquired immunodeficiency syndrome (AIDS). Over time, HIV infects and kills their CD4 T lymphocytes—immune system cells that stimulate B lymphocytes to make antibodies (proteins that recognize and destroy infectious agents) and that help CD8 T lymphocytes to kill cells that contain viruses and bacteria. The loss of CD4 T lymphocytes—a central player in “adaptive immunity”—leaves patients very susceptible to infections. However, the immune system does not die quietly. It does its best to fight HIV infection by mounting a cell-mediated immune response in which T lymphocytes attack HIV-infected cells. It also mounts a “humoral” immune response in which antibodies that recognize HIV are made. Some of these are neutralizing antibodies, which prevent HIV entering its host cells and replicating. Other antibodies may limit viral spread by inducing destruction of the virus. One way they can do this is by activating another part of the immune system called the complement system, which can break open and kill viruses (this is known as antibody-mediated complement lysis). In addition, antibodies and complement can coat the HIV virus particles so that phagocytes (for instance macrophages—yet another type of immune system cell) engulf and destroy the virus.

**Why Was This Study Done?:**

The role that humoral immunity plays in fighting HIV infection is complex and poorly understood. In particular, it is not clear whether the complement system helps to stop the spread of HIV or whether it inadvertently helps it to spread by facilitating its entry into host cells. It is important to understand as much as possible about the humoral immune response to HIV infection so that vaccines can be designed to provide maximum protection against HIV. In this study, the researchers have investigated whether antibody-mediated complement lysis controls the amount of virus in the blood of patients infected with HIV.

**What Did the Researchers Do and Find?:**

The researchers collected plasma (the liquid part of blood that contains circulating antibodies) from patients recently infected with HIV (acute infection) and patients who had been infected for some time (chronically infected). They also isolated HIV from each of the patients—so-called autologous virus. They then used a sensitive molecular biology assay to test each plasma sample for its ability to lyse the autologous virus (and also a standard virus) when supplied with complement from a healthy donor. Most of the plasma samples were able to lyse HIV, although the samples taken from chronically infected patients generally caused more lysis than those from acutely infected patients. In the chronically infected patients, the level of lysis induced was not related to the amount of virus in the patients' blood (viremia). However, plasma taken from acutely infected patients with higher viral loads was less active in the lysis assay than plasma taken from patients with lower viral loads. Finally, the researchers showed that the levels of antibodies in the various plasma samples to the two envelope proteins of HIV correlated strongly with the ability of each sample to lyse the standard virus and that these antibodies were mainly non-neutralizing antibodies.

**What Do These Findings Mean?:**

By showing that antibody-mediated complement lysis of HIV in the laboratory is inversely related to the patients' viral loads during acute infection, these findings suggest (but do not prove) that antibody-mediated complement lysis of HIV contributes to the control of viremia early in HIV infections. But, the importance of this form of humoral immunity in combating HIV infections remains uncertain, since complement has the potential to enhance as well as block viral spread. Further work is needed to unravel which of these effects is dominant in patients and to characterize fully the antibodies that activate complement. Nevertheless, the results of this study suggest that complement-activating antibodies should be considered in future attempts to design an effective HIV vaccine.

**Additional Information.:**

Please access these Web sites via the online version of this summary at http://dx.doi.org/10.1371/journal.pmed.0030441.

- National Institute of Allergy and Infectious Diseases fact sheet on HIV infection and AIDS
- US Department of Health and Human Services information on AIDS, including information on vaccines
- US Centers for Disease Control and Prevention information on HIV/AIDS
- Aidsmap information on HIV and the immune system provided by the charity NAM
- Wikipedia pages on the complement system (note: Wikipedia is a free online encyclopedia that anyone can edit)

## Introduction

The humoral immune response to HIV-1 infection is elicited early in infection and is generally vigorous at later disease stages, but its efficacy and modes in controlling viremia in vivo have not yet been completely unraveled. While numerous studies suggest that neutralizing antibodies may impact viral replication in vivo [1–7], the effect of antibodies in mediating effector functions and in limiting viral spread via the complement system, phagocytic cells, or killer cells remains uncertain. A detailed characterization and quantification of the relative contribution of direct and effector-mediated antibody functions on virus containment in vivo will be of central importance in defining relevant immune responses and designing vaccines.

The complement system is a key component of innate immune defense, and it provides a link to the adaptive immune response [8–10]. Besides inducing direct lysis of pathogens, the complement system also has opsonizing, phagocytosis-inducing, chemoattractant, and immune stimulatory functions [8,11]. Specific antibody alone can efficiently neutralize many viruses, but complement activation can enhance the antiviral effects of antibodies by opsonizing virions or inducing lysis of the particles [11]. Complement is effective in lysing many enveloped viruses, resulting in fragmentation of the outer membrane and disintegration of the nucleocapsid [11].

The role of complement in HIV pathogenesis appears to be multifaceted [12,13]. HIV-1 virions can activate complement via the classical pathway either by antibodies bound to the virus surface or, independent of antibody, by direct activation of complement through the viral envelope (Env) proteins gp41 and gp120 [14–17]. Direct lysis of HIV-1 upon antibody-mediated activation of the complement system has been demonstrated in vitro [18–20]. Complement has further been shown to boost the activity of neutralizing antibodies in vivo and in vitro [21,22], and non-neutralizing antibodies may play a role in containing viremia during the acute phase of infection by inducing direct complement-mediated virus lysis [23].

Opposing these observations, several reports have suggested that complement lysis of HIV-1 is limited in vivo by several host cell-derived complement control proteins incorporated by the HIV-1 virions [24–26]. Since antibody and complement efficiently lead to opsonization but not always to destruction of HIV-1, the virus may remain infectious [27]. In addition, opsonized virions were found to bind to complement receptor-expressing cells, which can enhance viral infectivity and transmission in vitro [12,13,28–31].

In the present study we sought to investigate the question of whether antibody-mediated complement lysis of HIV-1 contributes to virus containment in vivo and, if so, to quantify the relative contribution of this defense mechanism at different disease stages.

## Methods

### Patients and Virus Isolates

The study included 25 acutely infected and 31 chronically infected patients (Table 1). A control group of 11 healthy HIV-1 negative donors was recruited from volunteers affiliated with the University Hospital Zurich (Table S1). Patient demographics and selection and isolation of autologous virus have been described [32,33]. Written informed consent was approved by the ethics committee of the University Hospital Zurich and was obtained from all individuals (both infected and uninfected) according to the guidelines of the University Hospital Zurich.

### Plasma

Patient blood was sampled in Vacutainer tubes containing EDTA (BD [http://www.bd.com]), and plasma was collected within 6 h and frozen in 1 ml aliquots at −75 °C. Plasma was heat inactivated (1 h at 56 °C) to destroy complement activity and centrifuged at 500*g* for 10 min before use to remove cell debris and lipids. Normal plasma and infected-patient plasma were treated identically.

### HIV-1 Virion Complement Lysis Assay

A mix of sera from one to five healthy donors, stored at −75 °C, was used as source of complement.

**Figure 1:**
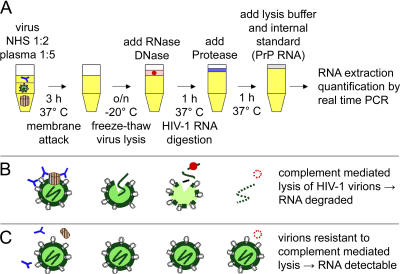
Schematic Overview of the Virion Lysis Assay (A) Primary HIV-1 virions were incubated with autologous plasma and complement and freeze-thawed once, and viral RNA was digested by RNase and DNase. After inactivation of RNase and DNase by protease and addition of an internal standard (PrP RNA), RNA was extracted and quantified by real-time PCR. (B) HIV-1 virions sensitive to antibody-mediated complement lysis were disrupted, making viral RNA accessible for degradation. (C) Viral RNA of complement lysis-resistant virions remained intact. RNA was extracted and could be quantified by real-time PCR.

HIV-1 virus stock (25 μl) was incubated with 20 μl of patient plasma (final dilution 1:5), 50 μl of complement (NHS final dilution 1:2), and 1 μl of RNase A (Qiagen, Valencia, California, United States) in RPMI 1640 (BioWhittaker [http://www.cambrex.com]) for a total volume of 100 μl; this mixture was incubated for 3 h at 37 °C (Figure 1). The reaction mixture was frozen at −20° C, thawed, and treated with RNase A (0.77 mg/ml, Qiagen) and DNase I (0.92 mg/ml) (Roche [http://www.roche-applied-science.com]) for 1 h at 37° C in a thermoshaker (Eppendorf; 1,400 rpm). Samples were treated with protease (0.71 mg/ml) (Qiagen) to remove RNase and DNase activity. Residual viral RNA in intact virions was extracted (RNeasy Mini Kit, Qiagen) and quantified by real-time PCR.

The extraction efficiency was controlled by adding and quantifying synthetic murine prion protein (PrP) mRNA (7,866 copies/μl) as an internal standard. In each assay, samples were tested in triplicate. Complement-mediated lysis activity was expressed as the percentage of lysed HIV-1 RNA copies compared to control plasma treatment. A mixture of plasma from 1–5 HIV-1-negative donors was used as negative control (no lysis activity, 0% value).

### Real-Time PCR

HIV-1 virions were quantified using primers either to Gag or to the 5′ end of HIV-RNA. For detection, dual-labeled fluorescent probes with a fluorescein (FAM) moiety at the 5′ ends and a tetramethylrhodamine (TAMRA) moiety at the 3′ end were used. HIV-Gag was measured using primers skcc1b [34] for cDNA synthesis, primers ts5′gag (upstream; 5′-CAAGCAGCCATGCAAATGTTAAAAGA-3′), boe2 (downstream), and boe3 (probe) [35] for amplification and detection. HIV-5′ RNA was measured using primer mf86 (5′-CCACACTGACTAAAAGGGTCTGAGGGATCT-3′), cr1 (5′-TCTCTGGCTAACAGGGAACCCACTGCTT-3′), cr2 (5′-TGACTAAAAGGGTCTGAGGGATCTCTAGTTACCAG-3′), and mf74tq (FAM-5′-AGCACTCAAGGCAAGCTTTATTGAGGC-3′-TAMRA). PrP mRNA was measured using PCR primers as previously described [36] and using a fluorescent probe prpe2+tq (FAM-5′-CAACCGAGCTGAAGCATTCTGCCT-3′-TAMRA). PCR was performed as described previously [37,38] in a single-tube system (Qiagen 1-step RT-PCR) with an additional “hot-start” using Ampliwax (Applied Biosystems, Foster City, California, United States) to separate cDNA synthesis and PCR amplification steps. cDNA synthesis and subsequent amplification were performed in duplicate in a real-time thermocycler (i-Cycler [BioRad, Hercules, California, United States]) as described [37,38].

### Anti-gp120, Anti-gp41, and Anti-p24 Plasma Antibody Titer

Plasma IgG titers to recombinant gp120 from the JR-FL strain (kindly provided by W. Olson, Progenics, Tarrytown, New York, United States), recombinant gp41 (amino acids 541–682 of the HxB2 strain [Viral Therapeutics, Ithaca, New York, United States]) and recombinant p24 (IIIB [Aalto BioReagents, Dublin, Ireland]) were determined by ELISA as described [32].

Plasma IgG titers to recombinant gp41 were determined as described for the other two antigens using plates coated with 0.1 μg of gp41 per well (amino acids 541 to 682 of the HxB2 strain; Viral Therapeutics). Maximal binding to gp41 was defined using the antibody 2F5 (kindly provided by H. Katinger) as a reference. Detection of bound antibody and calculation of midpoint titers were done as described [32].

### IgG Antibody Depletion

IgG was depleted from patient plasma using Protein G Sepharose beads (Amersham Pharmacia [http://www.amershambiosciences.com]). Beads were washed with PBS and incubated with patient plasma at room temperature for 1 h. Beads were removed by centrifugation and IgG-depleted plasma was taken from the supernatant.

### Neutralization Assays

Neutralization activity of patient plasma against replication competent autologous primary virus isolates was evaluated on peripheral blood mononuclear cells as described [39]. The plasma dilution over 1:40 causing 90% reduction (neutralization titer, NT_90_) in p24 production was determined by regression analysis.

Neutralization activity of patient plasma against the heterologous virus isolate JR-FL was evaluated on TZM-bl cells (National Institutes of Health AIDS Research and Reference Reagent Program) using JR-FL Env pseudotyped virus as described [4,40]. The plasma dilution over 1:20 causing 50% reduction (NT_50_) in luciferase reporter gene production was determined by regression analysis.

### Effects of Active Complement on Antibody-Mediated Inhibition of Viral Infectivity

Inhibitory activity of patient plasma in the presence of active or heat-inactivated complement was evaluated using JR-FL Env pseudotyped virus on TZM-bl cells [4,40]. Patient plasma (1:5 or 1:25 for acute and chronic patients, respectively) and complement in the same ratios as for the HIV-1 virion complement lysis assay were preincubated with virus (TCID_50_ = 1,000 ml^−1^) for 3 h at 37 °C without a subsequent freeze-thaw cycle (total volume, 60 μl). Plasma and complement content of the virus control were adjusted to the corresponding sample dilution with uninfected control plasma and active or inactivated complement, respectively. After preincubation, inhibition was measured in duplicates on TZM-bl cells at a final plasma concentration of 1:40 or 1:200 for acute and chronic patients, respectively.

### Statistical Analysis

Data analyses were performed using Prism version 4.03 for Windows (GraphPad Software, San Diego, California, United States) and Stata SE/9.2 for Windows (Stata Corporation, College Station, Texas, United States). The normality assumption was checked using the D'Agostino and Pearson omnibus normality test and had to be rejected for most variables (unpublished data). Hence, nonparametric methods were employed for testing of group differences (Mann-Whitney U test and Wilcoxon signed-rank test for unpaired and paired testing, respectively). Correlation analysis was performed using Spearman's rank correlation. All tests of significance were two-tailed and the level of significance was set at 0.05. P values reported in the main text and figures refer to values obtained after singular testing. Since multiple testing was performed caution must be taken in evaluating significance. In sum, 47 tests of significance were performed in our study, thus our level of significance was set at *p* < 0.00106 after Bonferroni correction to adjust for multiple testing. This stringent approach does not alter any of our main conclusions

## Results

### Antibodies in Patient Plasma Mediate Complement Lysis Activity against Autologous Viruses

**Figure 2:**
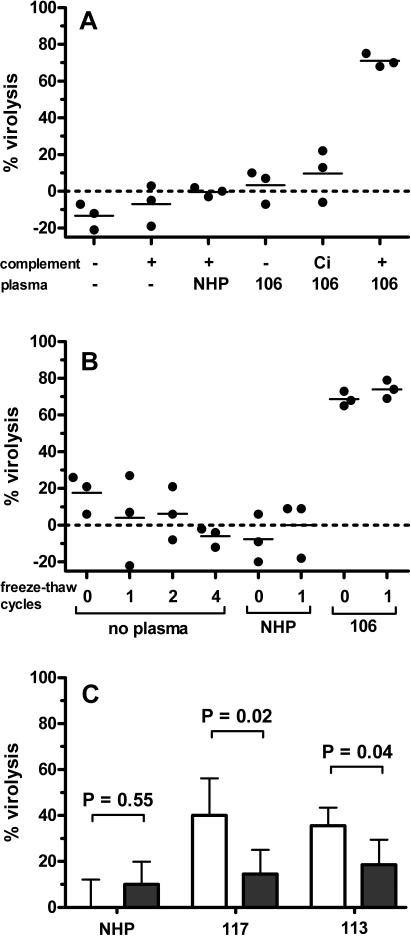
Virolysis by Antibody and Complement (A) Plasma from HIV-positive individuals specifically lyses HIV in presence of active complement. Virus isolate JR-FL was incubated with complement (+), without complement (−), or with inactivated complement (Ci) either in the absence of plasma (−) or with plasma from uninfected individuals (NHP) or from patient 106. One of three independent experiments is shown. (B) Up to four freeze-thaw cycles do not destroy intact HIV-1 virions. Virus JR-FL was incubated in the presence of active complement with medium (no plasma), with plasma from uninfected persons (NHP), or with plasma from patient 106. Reaction mixtures were subjected to the indicated number of freeze-thaw cycles and the effect on virus disintegration was measured. One of two independent experiments is shown. (C) IgG depletion reduces lysis activity. Plasma of uninfected individuals (NHP) and of patients 117 and 113 were depleted of IgG with Protein G Sepharose beads (darkened bars) and lysis activity was compared to untreated plasma (open bars). Error bars indicate standard deviation of triplicate measurements. Groups were compared using Mann-Whitney U test.

The primary intent of this study was to evaluate the influence of antibody- and complement-mediated lysis on viremia control in vivo. To this end we utilized an assay strategy that allows reliable and sensitive quantification of virus lysis by complement. In order to probe complement lysis activity under in vivo-relevant conditions, virus preparations used in our study were derived from infected primary peripheral blood mononuclear cells, because these cells are known to incorporate high numbers of complement control proteins, rendering these viruses less susceptible to complement lysis than those produced on immortalized cell lines [24]. In our assay, incubation of HIV-1 isolates with autologous patient plasma and complement is followed by one freeze-thaw cycle to completely disrupt the complement-attacked virions (Figure 1). Released RNA from lysed virions is digested by RNase treatment, and the RNase is inactivated by protease digestion. Viral RNA from the remaining intact virions is then extracted and quantified by real-time PCR. Using this assay scheme we were able to detect substantial complement lysis activity directed against the autologous virus in patient sera (Figure 2A). Patient plasma sampled at the time of virus isolation from a chronically infected individual (patient 106) induced lysis of 71% of the heterologous virus JR-FL in the presence of active complement. In the absence of active complement or of patient plasma, no efficient lysis was observed (lysis < 15%) (Figure 2A). Likewise, plasma from uninfected donors did not show specific lysis activity in the presence of active complement.

**Table 1:**
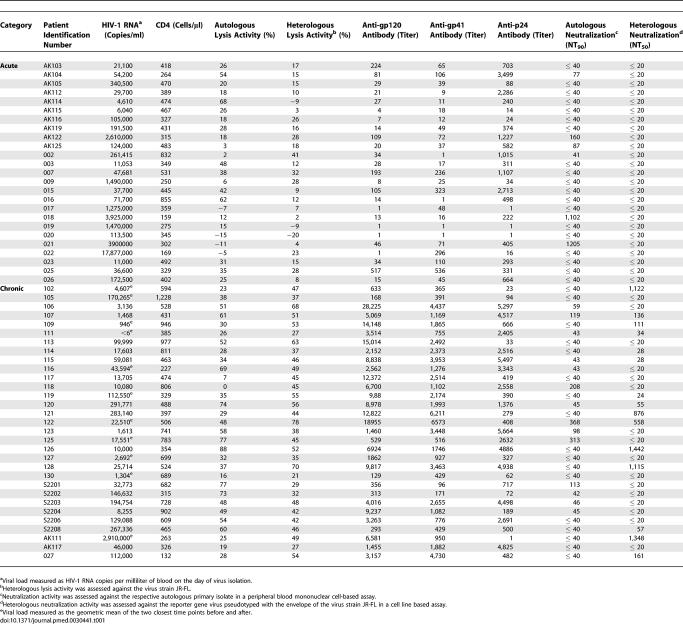
Patient Characteristics, Complement Lysis Activity, Antibody Titers, and Neutralization Titers

We further validated our assay and ensured that freeze thaw-cycles by themselves do not disrupt virions. As previously described, we found that neither the single freeze-thaw cycle used in our assay nor repeated cycles (2–4) led to disintegration of the virus in the absence of patient sera (Figure 2B) [41,42]. Irrespective of the isolate or plasma tested, a minimum of 10% of the virions appeared to be resistant to lysis. This resistant fraction may consist of viral particles carrying mutated, no, or low numbers of Env proteins, which could cause insufficient recognition by antibodies, or it may represent virus populations carrying high numbers of complement control proteins as suggested previously [24,43]. Neither increasing the plasma concentration nor blocking the complement control protein CD59 rendered this fraction of virions fully susceptible to lysis (unpublished data).

To determine if the observed virolysis activity depends on antibodies, we depleted plasma from two patients of IgG using Protein G Sepharose beads prior to incubation with virus and complement (Figure 2C). Undepleted plasma of patients 117 and 113 showed complement lysis activity of 40 and 36%, respectively. Depletion of IgG led to a substantial decrease of the observed lysis to 15% and 19% lysis for patients 117 and 113, respectively. The lysis activity observed after protein G treatment likely resulted from IgM antibodies and/or residual IgG. In summary, our data suggest that substantial complement-mediated lysis activity against HIV-1 virions may be present in a patient's autologous sera and that a considerable proportion of this lysis activity depends on IgG antibodies in a patient's plasma.

### Complement Lysis Activity in Plasma of Acutely and Chronically HIV-1-Infected Individuals against Autologous Virus

**Figure 3:**
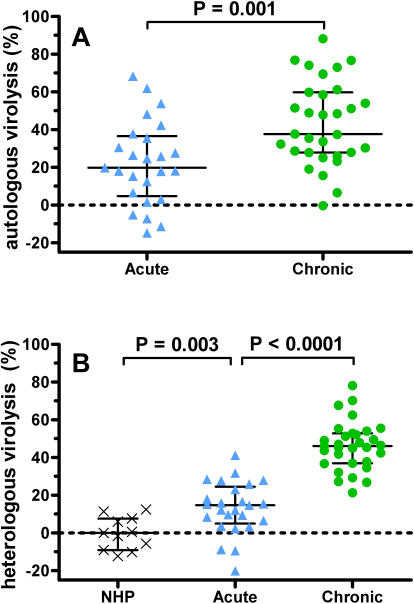
Lysis Activity against Virus in Plasma of Acutely and Chronically Infected Patients Complement-mediated lysis activity against autologous (A) and heterologous (JR-FL) (B) virus was compared between acutely (blue triangles) and chronically (green circles) infected individuals. HIV-specific lysis activity is present at acute and chronic diseases stages but generally higher during chronic infection. Crosses denote uninfected plasma controls. Groups were compared using Mann-Whitney U test.

To explore the impact of antibody-mediated complement virus lysis at different disease stages we measured patient plasma complement virolysis activity against autologous virus in a previously described cohort of 25 acutely and 31 chronically infected patients (Figure 3A and Table 1) [32,33]. Lysis activity against the autologous virus was measured in plasma samples derived at the time of virus isolation. The fact that the extent of lysis measured in our assay is independent of the amount of virus input (Figure S1) allowed us to compare the activities of multiple divergent virus isolates without normalizing virus input. The latter process can be prone to error, because absolute quantification of highly divergent virus stocks is subject to sequence variation and the resulting differences in RNA or p24 detection.

Virus input ranged from 4.8 ×10^3^ to 6.3 × 10^7^ viral RNA copies. In two patients with very high viral load (patients 018 and 022), virus detected in the plasma contributed over 1% to the total amount of HIV-1 copies measured in our assay. Therefore, patient plasma and heat-inactivated complement were used as negative control in these cases.

Autologous plasma induced complement lysis of the respective virus in the majority of infected patients (Figure 3A). Nineteen of 25 acutely infected and 30 of 31 chronically infected individuals showed specific lysis activity (over the 95% confidence interval of normal controls, i.e., more than 5.8% lysis). Complement lysis activity was highly variable between patients and ranged from 90% lysis to no lysis at a plasma dilution of 1:5. The median of complement lysis was almost twice as high during chronic disease stages (37.63% lysis) than during the acute phase (19.89% lysis, *p* = 0.001, Mann-Whitney U test) (Figure 3A and Table 2).

### Complement Lysis Activity in Plasma of Acutely and Chronically HIV-1-Infected Individuals against the Heterologous Strain JR-FL

Antibody responses to HIV-1, particularly during early disease stages, are thought to be predominantly strain specific. To investigate whether the antibodies that mediate complement lysis activity are specific for the autologous virus isolate or if cross-reactivity with heterologous isolates exists, we evaluated the capacity of our panel of patient plasmas to mediate lysis of the heterologous primary virus strain JR-FL. All plasma samples derived from chronically infected individuals, and 19 of 25 samples derived from acutely infected individuals mediated lysis of the virus strain JR-FL (Figure 3B). In agreement with the pattern seen for autologous virolysis, the median of lysis activity against JR-FL was almost three times as high in plasmas derived from chronically than from acutely infected patients (*p* < 0.0001, Mann-Whitney U test) (Table 2). In comparison, plasma from uninfected healthy controls (*n* = 11), induced no or only marginal lysis that was lower than lysis activity seen in the acute group (*p* = 0.003, Mann-Whitney U test) (Table 2), indicating that virolysis in infected patients is predominantly mediated by HIV-1-specific antibodies.

In a further analysis, autologous and heterologous lysis activities were assessed within each group and then separately in the combined cohort. In general, plasma lysis activity against heterologous and autologous viruses did not differ in the acutely or the chronically infected group. The analysis of the entire patient cohort also revealed no differences in lysis activity (Table 2). Although the overall pattern of reactivities against heterologous and autologous viruses was similar, with plasma from chronically infected patients inducing higher activities than did plasma from acutely infected individuals, we found no evidence for a correlation between the lysis activity against the autologous and heterologous virus strains (rho = 0.03, *p* = 0.87 for acute; rho = 0.19, *p* = 0.30 for chronic group) (Table 3), indicating that the type and specificities of antibodies mediating these activities may not completely overlap.

### Analysis of the Anti-Env and Anti-Gag Responses

**Figure 4:**
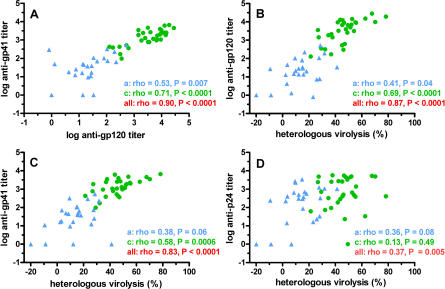
Correlation Analysis of Antibody Responses and Virolysis Activity (A) Correlation analysis of anti-gp120 and anti-gp41 antibody titers in plasma samples from the acute and chronic patient groups shows that titers to the envelope glycoprotein correlate tightly independently of disease stage. (B–D) Correlation analyses of heterologous lysis (JR-FL) with anti-gp120 (B) or anti-gp41 (C) revealed positive correlations, whereas anti-p24 (D) antibody titers did not associate with heterologous lysis. This suggests that lysis activity is driven by envelope-specific antibodies. Blue triangles denote acutely, green circles denote chronically infected patients. Spearman's rank correlation coefficient (rho) and *p*-values are depicted for the entire cohort (all), the acute patients (a), and the chronic patients (c). If antibody titers were below 1, the value 1 was used for statistical analysis.

In the course of HIV-1 infection, commonly a strong, high-titered antibody response to the structural viral proteins (Env and Gag) is elicited [44–46]. Complement lysis of HIV virions depends on antibodies that interact with the viral surface, and thus most likely involve antibodies that recognize the viral envelope proteins gp120 and gp41. It has been shown that the majority of Env-specific antibodies recognize epitopes that are inaccessible in the native oligomeric, fusion-competent form of the virus envelope [47–52]. Antibodies that bear neutralizing activity bind only to selected epitopes on the viral envelope oligomer that are involved in the interaction with the viral receptors or fusion. In contrast, antibodies that activate complement could be theoretically directed to any epitope accessible on the virion surface. To obtain an initial insight into which classes of anti-HIV antibodies mediate the complement lysis activity in patient plasma, we determined antibody responses to the viral core protein p24 and the Env proteins gp120 and gp41 with recombinant proteins derived from the viral strains JR-FL (gp120) and IIIB (p24 and gp41) (Table 1) [32]. As expected, all antibody responses were lower in acute than in chronic infection (Table 2) [32]. Since we had no recombinant gp41 from strain JR-FL available, we used the gp41 protein of the closely related IIIB strain (89.2% sequence homology at the amino acid level) to measure anti-gp41 responses in patient plasma. We found that anti-gp120 and anti-gp41 titers were associated in both the acute (rho = 0.53, *p* = 0.007) and the chronic patient groups (rho = 0.71, *p* < 0.0001) as well as when all patients were analyzed together (rho = 0.90, *p* < 0.0001), indicating that anti-gp41 and anti-gp120 responses may develop in parallel (Table 3 and Figure 4A). Anti-Env responses did not appear to be correlated with anti-p24 responses during the chronic phase (unpublished data), suggesting, as previously found [39], that anti-Env and anti-Gag responses are differentially regulated. During the acute phase, anti-gp120 and anti-p24 correlated significantly (rho = 0.76, *p* < 0.0001), probably reflecting the parallel maturation of the immune response against all epitopes during this stage.

### Impact of Anti-Env Responses on Complement Virolysis

In order to probe the impact of anti-Env antibody responses on viral lysis activity, we first analyzed the interdependency between lysis activity against JR-FL and antibody titers to the gp120 protein of this strain. We found a strong correlation between anti-gp120 titers and lysis activity when we analyzed the entire patient cohort (rho = 0.87, *p* < 0.0001) (Figure 4B and Table 3). This association between anti-gp120 titers and lysis activity was more pronounced in chronically infected individuals (rho = 0.69, *p* < 0.0001) than in acutely infected patients (rho = 0.41, *p* = 0.04). Likewise, we observed a strong association between JR-FL lysis activity and anti-gp41 antibody titers when all patients were analyzed together (rho = 0.83, *p* < 0.0001) (Figure 4C). When groups were analyzed individually, the association in the chronically infected patients alone was weaker (rho = 0.58, *p* = 0.0006) and no interdependency was evident in the acute infection group (rho = 0.38, *p* = 0.06). In contrast to the anti-Env responses, antibody titers to the core protein p24 exhibited no pronounced influence on the heterologous lysis activity induced during acute and chronic infection (rho = 0.37, *p* = 0.005 for the entire cohort) (Figure 4D). Taken together, our analysis strongly suggests that anti-Env responses are central in mediating complement lysis activity during both acute and chronic disease stages.

**Table 2:**
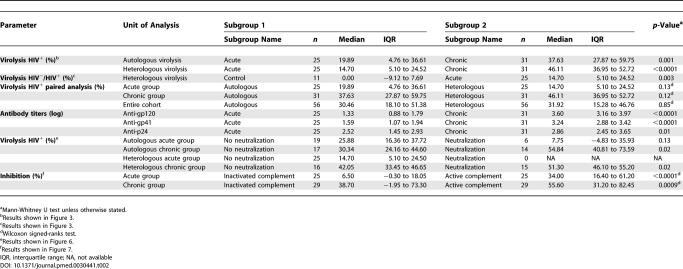
Group Comparisons of Measured Parameters

**Table 3:**
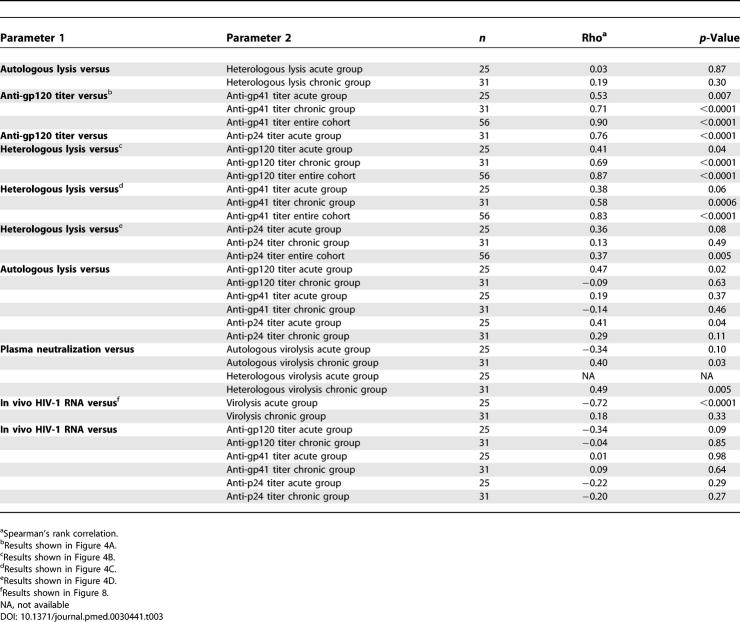
Correlation Analyses

Antibody titers against the autologous virus strains could not be determined. Due to the heterogeneity of virus isolates in our panel and the resulting variable sequence divergence in the recombinant proteins used to determine antibody titers, it was unlikely that we would see the same degree of association between autologous lysis activity and binding activity to the recombinant proteins. Nevertheless, a trend of a positive nonsignificant association between gp120 binding titers and lysis activity was observed in acutely infected individuals (rho = 0.47, *p* = 0.02). However, no evidence for an association was seen in chronically infected individuals (rho = −0.09, *p* = 0.63). Equally, no association between lysis activity to the autologous strain and reactivity to gp41 was seen in either patient group (rho = 0.19, *p* = 0.37 and rho = −0.14, *p* = 0.46 for the acute and chronic group, respectively). In accordance with the interdependency of anti-Gag and anti-Env responses during the acute phase, we observed a weak, nonsignificant correlation between p24 responses and the autologous lysis activity in acute patients (rho = 0.41, *p* = 0.04).

### Longitudinal Assessment of Autologous Complement Lysis Activity in Patient Plasma

**Figure 5:**
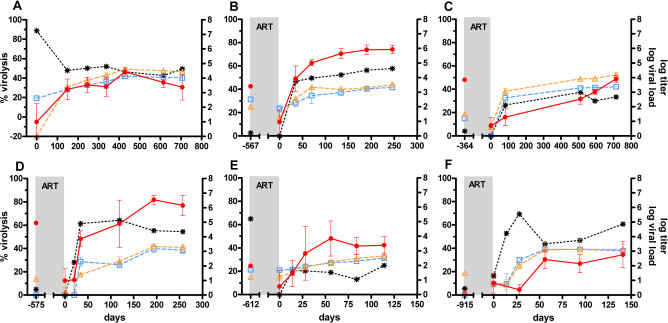
Longitudinal Assessment of Autologous Complement Lysis Activity in Patient Plasma Lysis activity against autologous virus (red circles) of plasma from six acutely HIV-1-infected patients was measured longitudinally and plotted against anti-gp120 titers (orange triangles), anti-gp41 titers (blue squares), and viral load (asterisks). At the first data point, patients were treatment-naïve and acutely HIV infected. All patients (except patient 022) subsequently went on antiretroviral therapy for the indicated time periods. Time point 0 was assigned to the date of treatment interruption and the remaining time points were calculated according to this time point. (A) Patient 022 (treatment-naïve), (B) patient 015, (C) patient 003, (D) patient 016, (E) patient 026, and (F) patient 002.

Our cross-sectional analysis suggested that complement lysis activity is mediated by anti-Env responses and may increase when the antibody response broadens. To investigate the development of the lysis activity more closely, we monitored antibody responses and autologous complement lysis activity over extended time periods in plasma of six acutely HIV infected patients (Figure 5). Of these six individuals one stayed treatment-naïve (patient 022). The remaining five patients initiated antiretroviral treatment during the acute infection phase and subsequently stopped treatment after 12–31 months on successful antiretroviral treatment. Lysis activity was measured against the autologous strain derived at the first specimen collection. Antibody reactivity and lysis activity were subsequently measured solely during the treatment-free periods.

**Figure 6:**
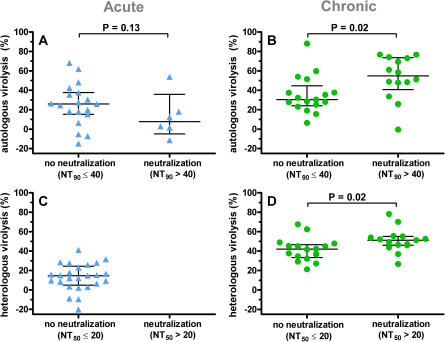
Neutralizing Antibodies Are Not the Major Constituent of Complement Lysis-Inducing Antibodies Complement lysis activity of non-neutralizing and neutralizing patient plasmas was compared in the acute and the chronic infection cohorts. Autologous neutralization (A and B) and heterologous (JR-FL) (C and D) neutralization are shown, with blue triangles denoting acutely, green circles chronically infected patients. Each data point represents the mean of two or three independent experiments with plasma from the same patient. Groups were compared using Mann-Whitney U test.

As expected, titers of antibody to gp120 and gp41 increased steadily in all patients, which was paralleled by an increase in lysis activity. In the five patients who underwent treatment interruption it is evident that along with viral load levels, antibody responses and lysis activity rose. A direct impact on in vivo viral load levels cannot be easily investigated in this setting, since upon rebound it takes several weeks to months before set points of viral load and immune responses are reached. Collectively, our observation strongly suggests that early antibody responses against the viral Env proteins gp41 and gp120 mediate lysis activity against the autologous virus strain.

### Plasma Neutralization Activity

Neutralizing antibodies directed against the autologous HIV strain can appear during the acute infection phase [4,5], but are in general more pronounced during chronic disease stages [1,6]. A central question in our analysis, therefore, was to investigate whether neutralizing antibodies are elicited alongside complement activating antibodies or if the latter precede the neutralizing response. To this end we evaluated the neutralizing activity mediated against autologous and heterologous virus in our patient cohort.

Only six (24%) of the plasmas from the 25 acutely infected patients and 14 (45%) from the group of 31 chronic patients showed measurable autologous neutralization activity (NT_90_ > 40) (Figure 6A and 6B). A slightly higher autologous lysis activity was observed among chronically infected patients whose plasma had neutralizing activity against the autologous viruses, although this difference did not reach significance (*p* = 0.02, Mann-Whitney U test). No difference was seen in the acute group (*p* = 0.13) (Table 2). Likewise, we observed no correlation between autologous lysis and neutralization activity in either group (Table 3). The latter could imply that non-neutralizing antibodies contribute predominantly to the lysis activity. However, we cannot exclude the possibility that neutralizing antibodies are present at concentrations below the detection level of the neutralization assay and that low concentrations of these antibodies could suffice to induce virolysis. Nevertheless, neutralizing activity per se is low at best in these patients and thus is unlikely to drive viremia control.

Although none of the patient plasmas from the acute group showed neutralizing activity against the heterologous virus strain JR-FL (Figure 6C), 15 (48%) of the chronically infected patients neutralized this virus (NT_50_ > 20) (Figure 6D). Heterologous lysis activity was again higher among chronically infected patients with neutralizing activity (*p* = 0.02, Mann-Whitney U test) (Table 2). A marginal correlation between heterologous lysis and neutralization activity was observed in the chronic group (rho = 0.49, *p* = 0.005) (Table 3), indicating that antibody reactivities involved in neutralization and lysis of heterologous HIV-1 virions may overlap to some extent.

### Complement Lysis Leads to Reduction in Viral Infectivity

**Figure 7:**
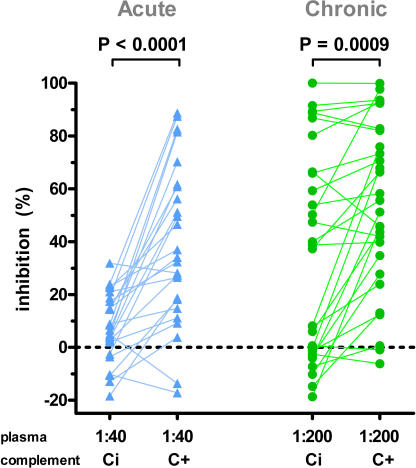
Influence of Complement on Viral Infectivity In Vitro Inhibitory activity of patient plasma against the heterologous virus JR-FL was measured in presence of active (C+) or heat-inactivated (Ci) complement on TZM-bl cells. Blue triangles denote acutely, green circles chronically infected patients. Data points are means of two independent experiments with plasma from the same patient. Differences in inhibition between inactivated and active complement within the subgroups were compared using Wilcoxon signed-rank test. The results demonstrate that complement increases the inhibitory activity of HIV specific antibodies in vitro.

Since our virus lysis assay measures complement destruction under nonphysiological conditions (freeze-thaw cycle and RNA digestion) we investigated whether virion lysis occurs also under natural conditions and leads to a reduction of viral infectivity. To circumvent nonspecific inhibitory or enhancing effects of human plasma in our in vitro assay, we chose assay conditions in which controls contained the corresponding concentration of normal human plasma and complement. To be able to compare effects of antibodies in the absence of active complement (neutralization) and inhibition induced in the presence of active complement (neutralization and complement lysis), we chose plasma dilutions that allowed simultaneous evaluation of both effects. Thus, patient plasmas from the acute group, all of which had marginal neutralization activity against the isolate JR-FL, were studied at a dilution of 1:40; chronic patients, whose plasma generally had higher neutralization activity, were studied at a dilution of 1:200. Each patient group was assessed separately, which allowed us to use two different plasma dilutions. The latter was necessary, because otherwise neutralization activity would have dominated the readout in the chronic group. In the majority of patients the presence of active complement increased the inhibitory effect of the patient plasma (Figure 7). Median inhibition was markedly lower when complement was inactivated, both in the acute (*p* < 0.0001, Wilcoxon signed-rank test) (Table 2) and the chronic group (*p* = 0.0009), demonstrating that complement lysis reduces viral infectivity.

### Impact of Complement Lysis on Viremia Control In Vivo

Both HIV-1-specific T cell activity and—somewhat delayed—humoral immune responses develop early in HIV infection. The initial rise in cytotoxic T lymphocytes responses is often associated with a decline in viremia shortly after infection [53]. Although neutralizing activity of antibodies in patient sera during the acute phase can be found [4,5], it is not clear to what extent these antibodies contribute to viral containment. Even less is known about the effects of complement lysis mediating antibodies in vivo. Since our analyses thus far had clearly shown that complement-activating antibodies are elicited soon after infection, we next investigated whether these antibodies have a clear impact on viremia control in vivo. To this end we probed whether interdependencies existed between autologous or heterologous lysis activities and the plasma viral load measured at the time of plasma and virus collection.

**Figure 8:**
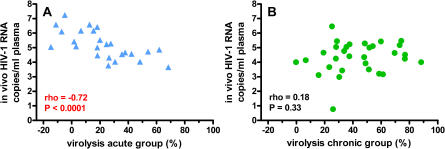
Correlation Analysis of Complement Virolysis and In Vivo Viremia Levels In vitro-determined autologous virolysis activity was correlated with in vivo HIV-1 RNA copies measured per milliliter of plasma in the acutely infected group (A) and the chronically infected group (B). Correlations were evaluated using Spearman's rank correlation. Our data demonstrate that increased antibody-mediated complement lysis coincides with lower viral loads in the acute phase. No evidence was found for a similar correlation in the chronically infected group.

We found that lysis activity against autologous plasma inversely correlated with the in vivo viral load (RNA copies/ml of plasma) in the acute group (rho = −0.72, *p* < 0.0001), but not in the chronic group (rho = 0.18, *p* = 0.33) (Figure 8 and Table 3). Our assessment of the in vivo activity of the viral lysis activity solely focuses on the potential contribution of the HIV-specific antibodies in the patient plasma. In vivo lysis activity may further vary due to variations in complement activity influenced by different genetic backgrounds and disease progression. No interdependency between CD4 levels and virolysis activity or virus load existed in the acute cohort (unpublished data). In contrast to autologous lysis activity, heterologous lysis activity and viral load did not correlate, indicating that isolate-specific antibodies may dominate virolysis activity in vivo. Antibody titers to the viral proteins gp120, gp41, and p24 showed no correlation with the viral load in both the acute and the chronic cohorts (all rho values ≤ 0.34, *p* ≥ 0.09) (Table 3). Hence, the inverse association between autologous lysis activity and viral loads during the acute phase does not simply reflect the appearance of binding antibodies during this period in these patients. Taken together our data demonstrate that increased antibody-mediated complement lysis coincides with lower viral loads in the acute phase. Therefore, complement lysis could potentially function as an early immune defense mechanism against HIV-1 that impacts on viremia control in the acute infection phase, during which the adaptive immune response has not yet fully matured.

## Discussion

In recent years substantial effort has been put into investigating the humoral immune response to HIV-1. While neutralizing antibodies are considered a correlate of protection against HIV-1 and a necessary component of vaccine-induced immune responses, the role of effector mechanisms mediated by anti-HIV antibodies in immune control remains largely unclear. In the present study we investigated the efficacy of the humoral immune response elicited during acute and chronic disease stages in inducing complement-dependent lysis of HIV virions. Evidence obtained through a novel complement virion lysis assay suggests that antibody-mediated complement lysis in the plasma of HIV-1-infected individuals has been underestimated in the past. Previously used virolysis assays mostly relied on the measurement of reverse transcriptase [54] and p24 antigen released from lysed virions [24,25,55], which limited their use in measuring complement lysis activity directly in patient plasma because antibodies specific for reverse transcriptase and p24 can interfere with the detection of these proteins following virolysis. The development of a novel, highly sensitive and quantifiable assay for virolysis-mediating antibody responses in plasma samples was thus key for the current study, and it allowed us to investigate complement lysis in patient samples directly by quantifying viral RNA.

We found that, in most patients, antibodies are elicited very early after infection (< 3 mo) that induce complement-mediated lysis of the autologous virus and thus could contribute to viremia control during the acute phase of HIV-1 infection. The latter is signified in our study by a tight inverse correlation between lysis activity and in vivo-measured viral loads. Our results corroborate the findings by Aasa-Chapman and coworkers, who recently reported complement lysis activity in sera of some acutely infected individuals [23]. In our study, lysis activity was in general higher during chronic infection, probably reflecting the elevated antiviral antibody responses in later stages of the infection. Unlike in acute infection, however, no association between complement lysis activity and the viral set point was seen in chronically infected patients. Virolysis, if active in vivo, should destroy virions in both disease stages and even more so during chronic infection, as the in vitro-measured activity is higher then. A possible explanation of these contrasting observations in acute and chronic infection is that complement lysis-activating antibodies may be important only early in infection, when other adaptive immune responses have not yet fully matured. In chronic infection the overall influence of these complement-activating antibodies may be still present, but their impact lower or negligible because neutralizing antibody and cellular immune responses have matured. Thus the effect of virolysis on viral load levels may only be measurable during the acute phase, because during later stages of the infection other immune functions have developed that are more powerful and therefore determine the viral set point. Virolysis activity, while still present, would then have a comparatively small effect on in vivo virus loads.

In support of the differences between acute and chronic group we found that, particularly during the acute phase, complement lysis appeared to be predominantly mediated by non-neutralizing antibodies. Lysis activity against the heterologous strain JR-FL was observed despite the absence of neutralizing antibodies against this virus in acutely infected patients. Likewise, lysis and neutralization activity against the autologous viruses showed no interdependency. Although in the chronic cohort higher lysis activity was found in patients that harbored neutralizing activity, we observed no direct relationship of these reactivities against the autologous virus, and only a minor influence in the heterologous system. Nevertheless, we cannot exclude the possibility that neutralizing antibodies at concentrations lower than the detection limit of the neutralization assay are present and contribute to virolysis activity.

The role of complement in HIV pathogenesis has been a matter of debate for many years. While several reports have proposed that complement-dependent virus lysis occurs in vivo [43,56], and that complement can boost the effect of neutralizing antibodies both in vivo and in vitro [21–23], other groups have demonstrated a pronounced resistance of HIV to complement lysis in vitro [24–26,55,57,58]. Moreover, opsonization of virus with antibodies and complement has been shown to enhance viral infectivity and transmission in vitro [12,13,28–31]. Which of these complement activities occur in vivo and what their relative contributions are in inhibiting or enhancing viral replication, remain currently unknown. Whether complement lysis activity is outweighed by more specific adapted immune responses in chronic infection, as suggested above, or whether at later disease stages enhancing effects of antibodies and complement emerge and reduce the impact of lysis activity are questions bearing further investigation.

The factors that direct complement action to either lysis or enhancement have not been fully defined. While our study demonstrates inhibitory effects of complement and antibody in early HIV infection, complement-dependent enhancement of HIV infection of complement receptor-bearing cells likely occurs as well in vivo. Complement concentration may vary at different disease stages [43,59–62]; thus, in vivo, individual lysis activity could differ due to fluctuations in complement supply.

Moreover, complement concentrations in tissue are only in the range of 10%–20% of the levels in serum [63–65]. Thus the relative contribution of virus lysis and enhancement in tissue and in the periphery may differ and needs to be investigated. Further studies will be needed to unravel whether the beneficial or the detrimental effects of complement and antibody dominate in vivo.

It is currently also not known whether antibodies that mediate complement lysis and enhancement are directed to the same epitopes. Nevertheless, it seems feasible that their reactivities overlap to some extent. Our data indicate that if enhancing activity impacts on virus replication in vivo, it appears not to be a direct consequence of high antibody titers, as we observed no notable interdependency between binding antibody titers to HIV and viral loads. We demonstrated, however, that also under culture conditions, complement in concert with HIV-1-specific antibodies reduces viral infectivity, which further supports a role for virus lysis in vivo.

We determined in our study that complement lysis activity is mediated by anti-Env antibodies, as demonstrated by the tight correlation between lysis activity and antibody titers to gp120 and gp41. While neutralizing antibodies are known to recognize native viral Env oligomer epitopes that are involved in receptor binding and fusion, complement-activating antibodies are not limited to these sites. Additionally, non-neutralizing antibodies binding to the oligomer, or antibodies reacting with gp120 monomers or with epitopes on gp41 that are exposed after shedding of gp120, could potentially activate complement if bound to the virion in sufficient densities.

A more detailed characterization of the antibodies mediating lysis activity will be particularly important if complement-activating antibodies are to be considered a component of effective vaccines.

Our finding that HIV-1 is susceptible to lysis mediated by specific antibodies in patient sera and complement is in agreement with a previous study by Sullivan and coworkers, who showed that virus derived from patient plasma can be lysed in the presence of complement due to virion-bound antibodies that activate the complement system [43]. While this study provided initial evidence that complement lysis of HIV-1 may occur in vivo, it was limited to the analysis of patient samples with high viral load due to the inherent insensitivity of the assay used. The range of lysis activity found in this ex vivo analysis (14%–86% lysis) corresponds closely with the values obtained in our study. The profound complement lysis seen in our study and, most importantly, the inverse correlation between lysis activity and in vivo viral loads during the acute phase strongly suggest that antibody-mediated complement lysis could contribute to viremia control and may therefore be a defense mechanism in vivo.

Several recent reports have emphasized that complement activation boosts humoral and cellular immune responses [8]. Consequently, complement-stimulating antibodies, besides mediating direct lysis of virions, might have an important function in aiding the development of immune responses to HIV both in natural infection and in responses to vaccines.

While our study demonstrates that presence and magnitude of autologous antibody-mediated complement lysis of HIV-1 coincide with increased viremia control during the acute infection phase, direct associations cannot be formally proven. Immune functions and the timing of their appearance during the course of early HIV-1 are intertwined, and it is therefore difficult to ascertain direct relationships. Activities of cytotoxic T cells, neutralizing antibodies, antibodies that mediate antibody-dependent cellular toxicity, opsonization, aggregation, phagocytosis and—as our current report suggests—antibodies that mediate virus lysis via activation of the complement system will impact on viral spread in vivo. Total or neutralizing antibody titers or CTL activity, however, have not been proven to reliably predict viremia levels in the past [39,66–69]. While cellular immune responses were not assessed in our study, the fact that virolysis activity induced by complement and antibody inversely correlated with in vivo viral loads, but not with binding antibody titers per se nor with neutralizing activities, leaves room for a scenario in which virolysis has an impact on viral load levels in vivo. Nevertheless, subsequent studies will be required to unravel the exact associations between these diverse immune functions and to assess their individual impact. Based on our current study, we hypothesize that complement lysis activity induced by specific antibodies may be an additional player in the network of immune function countering HIV replication in vivo and that, therefore, the impact of these antibodies in vivo should be further evaluated, as they could be a critical component of vaccine-induced immunity to HIV-1.

## Supporting Information

Figure S1 The Extent of Lysis Is Independent of the Amount of Virus Input The varying amounts of RNA input into the autologous assays were correlated with measured autologous virolysis activities. Correlation was evaluated using Spearman's rank correlation (*n* = 56, rho = −0.09, *p* = 0.53, two-tailed).
(841 KB DOC)

Table S1 Control Group Demographics (35 KB DOC)

## Abbreviations

NT: neutralization titer
