# Supplementary material for: Complement Lysis Activity in Autologous Plasma Is Associated with Lower Viral Loads during the Acute Phase of HIV-1 Infection
Source: PLoS Med. 2006 Nov 21;3(11):e441. doi: 10.1371/journal.pmed.0030441 (PMC1637124; doi:10.1371/journal.pmed.0030441)
Supplement: Supplementary file 2 [file pmed.0030441.st001.doc]

Table S1: Control group demographics

| **code** | **gender (m/f)** | **age (years)** |
| --- | --- | --- |
| co 1 | f | 28 |
| co 2 | f | 40 |
| co 3 | m | 50 |
| co 4 | m | 28 |
| co 5 | f | 29 |
| co 6 | m | 34 |
| co 7 | f | 40 |
| co 8 | m | 43 |
| co 9 | m | 28 |
| co 10 | m | 43 |
| co 11 | m | 39 |
| **11** | **7/4** | **Median 39** |
|  |  | **IQR 28 - 43** |
